# Recent NICE guidance of interest to surgeons

**Published:** 2013-03

**Authors:** Bruce Campbell

## Quality standards

### Colorectal cancer (QS20, August 2012)

Quality standards are a set of statements about measurable components of high quality and cost effective care. They are based on consideration of effectiveness, patient safety and patient experience. Quality standards are designed to be aspirational but achievable. Commissioners will use them in setting contracts.

The quality standard on colorectal cancer was published in August 2012 and has eight key statements:

*Statement 1*: People with suspected colorectal cancer without major co-morbidity are offered diagnostic colonoscopy.

*Statement 2*: People with colon cancer are offered contrast enhanced computed tomography (CT) of the chest, abdomen and pelvis to determine the stage of the disease.

*Statement 3*: People with rectal cancer are offered contrast enhanced CT of the chest, abdomen and pelvis to determine the stage of the disease, and pelvic magnetic resonance imaging to assess the risk of local recurrence.

*Statement 4*: People with rectal cancer are offered a preoperative treatment strategy appropriate to their risk of local disease recurrence.

*Statement 5*: People with locally excised, pathologically confirmed stage I colorectal cancer whose tumour had involved resection margins (<1mm) are offered further surgery or active monitoring.

*Statement 6*: People with contrast enhanced CT of the chest, abdomen and pelvis suggesting liver metastatic colorectal cancer have their scans reviewed by the hepatobiliary multidisciplinary team to decide whether further imaging is needed to confirm suitability for surgery.

*Statement 7*: People with locally advanced or metastatic colorectal cancer whose disease progresses after first-line systemic anticancer therapy are offered second-line systemic anticancer therapy if they are able to tolerate it.

*Statement 8*: People free from disease after treatment for colorectal cancer are offered regular surveillance.

The full text of the quality standard provides more detail about what each statement means for different audiences, including clinicians, patients and commissioners. It also provides links to the evidence underpinning the statements and to related guidance.

## Medical technologies

### Mega Soft Patient Return Electrode for use during monopolar electrosurgery (MTG11, August 2012)

This is about ‘diathermy’! The Mega Soft (Megadyne, Draper, UT, US) system replaces a standard adhesive monopolar diathermy plate with a pressure relieving mattress that remains on the operating table and is cleaned between patients. It contains an electrode that has much more extensive contact with the patient than a standard adhesive pad.

The evidence supported the claim that Mega Soft may offer advantages for some patients by: (a) avoidance of shaving for application of adhesive pads and (b) avoidance of adhesive for those with fragile or damaged skin. Use of Mega Soft also reduces the risk of burns, provided other aspects of operating theatre practice are of a good standard. In addition, there may be advantages to operating theatre staff in terms of increased convenience and reduced setting-up time.

Despite these potential advantages, cost modelling did not show any likely financial savings as a result of using the Mega Soft system: it was likely to be cost neutral. Two factors that influenced this modelling were: (a) the estimate that, in practice, only about 20–30% of all patients need to be shaved for application of adhesive diathermy pads and (b) the recognition that in day case operating theatres patients remain on the same trolley for anaesthesia, surgery and recovery so two Mega Soft mattresses would be required for each theatre (in contrast to inpatient operating theatres, where only one mattress would be needed). The guidance recommends that clinicians and managers consider these factors in judging the likely benefits of adopting Mega Soft in their operating theatres.

## Clinical guidelines

### Lower limb peripheral arterial disease (CG147, August 2012)

This guideline covers a wide range of issues relating to the management of patients with intermittent claudication and with more severe ischaemia, in primary and secondary care. Most of the recommendations describe well-established practice but some are likely to stimulate discussion among vascular specialists, regarding their implications and their implementation. These include:

*Provision of information for patients about how to manage pain and about how to access support for depression and anxiety*: Information for these patients has been offered largely by specialists in secondary care but pain control and counselling/psychiatric support services tend to fall more in the province of primary care. This recommendation seems intended to encourage the provision of more information for these patients in primary care.

*Measurement of ankle–brachial pressure index in all patients*: This is important for primary care as an aid to referral. In secondary care, many vascular specialists depend on their experience in interpreting Doppler sounds rather than necessarily measuring pressures. The recognition that some patients (especially diabetics) can have significant ischaemia with an ankle–brachial pressure index of >1 is very important: failure to appreciate this can result in detrimental delay in referral.

*The recommendation that duplex should be the first-line imaging method, followed by magnetic resonance angiography if revascularisation is being considered*: **This may not concur with the selective approach to imaging that some vascular specialists favour (eg in patients with severe ischaemia and a good femoral pulse, for whom femoral arteriography accompanied by any feasible balloon angioplasty may be judged preferable to two prior imaging sessions).

*Provision of supervised exercise classes for all patients with intermittent claudication*: This poses practical problems in a health service where still only a minority of cardiac patients has access to exercise classes as part of their rehabilitation. The question of funding is central to this: whether by primary care, secondary care or patients paying for themselves.

## Interventional procedures

### Partial replacement of a meniscus of the knee using a biodegradable scaffold (IPG430, July 2012)

Traditionally, severe damage to a meniscus in the knee joint has been treated by partial meniscectomy. Meniscal repair is possible only in a minority of patients. Partial replacement of a meniscus of the knee using a biodegradable scaffold aims to provide a matrix for cell adhesion and vascular ingrowth after removal of the damaged parts of the meniscus. A peripheral rim of meniscus is left; the implant is trimmed to shape and sutured to it. Implants may be made of polyurethane or collagen. Two main patient groups are likely to be considered for implants: young active patients and older patients with meniscal degeneration.

A randomised controlled trial (RCT) of 311 patients reported no significant differences in pain scores between patients treated by partial meniscectomy either with or without an implant after five years. Reoperation rates at five years were 10% and 23% respectively (significance not reported). A smaller RCT of 60 patients had a shorter follow-up duration with a high dropout rate and reported no outcomes to support any significant advantage of the procedure. A non-randomised study documented significantly better quality of life scores after 11 years in 17 patients who received implants compared with 16 treated by medial meniscectomy alone. Case series data showed the need for another surgical procedure in 9 of 52 patients (17%). Adverse events were infrequent: four patients with implants developed swelling, effusion and redness, compared with one patient who had a partial meniscectomy alone, in the RCT of 311. A case series of 25 patients described one instance of infection and one of mechanical failure during 10–13 years of follow-up.

Based on this evidence, it was judged that there are no major safety concerns about this procedure but the evidence on efficacy (short-term symptom relief and reducing the need for further operations in the long term) was seen as limited. The guidance therefore recommends use only with ‘special arrangements’ for governance, consent and audit. It recommends particularly that patients should understand the uncertainties regarding any long-term advantage and also the need for prolonged rehabilitation after the procedure. Further research and data collection were encouraged.

